# Determinants of survival following heart transplantation in adults with congenital heart disease

**DOI:** 10.1186/s13019-024-02509-0

**Published:** 2024-02-10

**Authors:** Hüseyin Sicim, Pierre Emmanuel Noly, Suyash Naik, Vikram Sood, Richard G. Ohye, Jonathan W. Haft, Keith D. Aaronson, Francis D. Pagani, Ming-Sing Si, Paul C. Tang

**Affiliations:** 1 Department of Cardiac Surgery, University of Michigan Frankel Cardiovascular Center, Ann Arbor, MI USA; 2 Department of Cardiovascular Surgery, Mayo Clinic, Rochester, MN USA; 3 Department of Cardiac Surgery, Université de Montréal, Montréal, QC Canada; 4 Department of Internal Medicine, Division of Cardiovascular Medicine, University of Michigan Frankel Cardiovascular Center, Ann Arbor, MI USA; 5 Department of Surgery, Division of Cardiac Surgery, University of California Los Angeles, Los Angeles, CA USA

**Keywords:** Heart transplant, Heart failure, Congenital heart disease, Outcomes

## Abstract

**Background:**

Adult patients surviving with congenital heart disease (ACHD) is growing. We examine the factors associated with heart transplant outcomes in this challenging population with complex anatomy requiring redo-surgeries.

**Methods:**

We reviewed the United Network for Organ Sharing-Standard Transplant Analysis and Research database and analyzed 35,952 heart transplants from January 1st, 2000, to September 30th, 2018. We compared transplant characteristics for ischemic cardiomyopathy (ICM) (n = 14,236), nonischemic cardiomyopathy (NICM) (n = 20,676), and ACHD (n = 1040). Mean follow-up was 6.20 ± 4.84 years. Kaplan–Meier survival curves and Cox-proportional hazards analysis were used to analyze survival data.

**Results:**

Multivariable analysis confirmed that ACHD was associated greater in-hospital death compared to ICM (HR = 0.54, *P* < 0.001) and NICM (HR = 0.46, *P* < 0.001). Notable factors associated with increased mortality were history of cerebrovascular disease (HR = 1.11, *P* = 0.026), prior history of malignancy (HR = 1.12, *P* = 0.006), pre-transplant biventricular support (HR = 1.12, *P* = 0.069), postoperative stroke (HR = 1.47, *P* < 0.001) and postoperative dialysis (HR = 1.71, *P* < 0.001). ACHD transplants had a longer donor heart ischemic time (*P* < 0.001) and trend towards more deaths from primary graft dysfunction (*P* = 0.07). In-hospital deaths were more likely with ACHD and use of mechanical support such as use of right ventricular assist device (HR = 2.20, *P* = 0.049), biventricular support (HR = 1.62, *P* < 0.001) and extracorporeal membrane oxygenation (HR = 2.36, *P* < 0.001). Conditional survival after censoring hospital deaths was significantly higher in ACHD (*P* < 0.001).

**Conclusion:**

Heart transplant in ACHD is associated with a higher post-operative mortality given anatomical complexity but a better long-term conditional survival. Normothermic donor heart perfusion may improve outcomes in the ACHD population by reducing the impact of longer ischemic times.

**Supplementary Information:**

The online version contains supplementary material available at 10.1186/s13019-024-02509-0.

## Introduction

The most common congenital anomalies diagnosed at birth are congenital heart diseases (CHD) [1] and it affects about 1% of the ~ 40,000 births per year in the United States [2]. Modern advances in the surgical repair and management these defects in early life have allowed ~ 85–90% of children born with CHD to reach adult age [3, 4]. This achievement resulted in a large adult congenital heart disease (ACHD) population with congenitally corrected and/or palliated congenital cardiac conditions who need heart transplants [5]. The International Society for Heart and Lung Transplantation (ISHLT) 2018 registry reported that 3% of adult heart transplants between 2009 and 2017 were for ACHD [6] which is an increase from 2.7% between 2004 and 2008 [7]. Importantly, most transplant candidates with ACHD have single ventricles, a subgroup that is significantly more challenging than those with biventricular physiology [8].

Given the growing population of ACHD transplant candidates and the complexity of this patient population, identifying key outcomes determinants is critical for improving transplant outcomes. We examine the national Unified Organ Sharing Network (UNOS) database in detail to develop an understanding of the drivers of patient prognsosis and formulate management strategies to optimize transplant outcomes.

## Patients and methods

### Study population and data

We reviewed the United Network for Organ Sharing-Standard Transplant Analysis and Research (UNOS-STAR) database and analyzed 35,952 heart transplants from January 1st, 2000, to September 30th, 2018. For patients undergoing heart transplant for ischemic cardiomyopathy (ICM) (n = 14,236), nonischemic cardiomyopathy (NICM) (n = 20,676), and ACHD (n = 1,040), we compared recipient and donor characteristics as well as outcomes. The mean follow-up for the total study population was 6.20 ± 4.84 years. The University of Michigan institutional review board approved this study (IRB#HUM00194249).

### Statistical methods

Categorical variables were compared using Pearson chi square test or Fisher’s exact test. Continuous variables were analyzed with Student’s *t* test or Analysis of variance (ANOVA) for more than 2 groups. Recipient demographics, clinical characteristics, hemodynamics, presentation characteristics and concomitant transplants as well as donor age, clinical features and blood type were analyzed using Cox proportional hazards to determine variables influencing survival. Univariable and multivariable forward and reverse logistic regression was used to evaluate for factors associated with mortality. Kaplan–Meier Survival analysis with Log-Rank statistics was also used to analyze survival data. Conditional survival based on survival to hospital discharge were also examined. A *P* value < 0.05 was considered statistically significant. Statistical analysis was performed using the Statistical Package for the Social Sciences software (SPSS Inc., Chicago, IL).

## Results

### Baseline characteristics

Compared to the ICM and NICM groups (Table 1), ACHD transplant recipients were younger (35.49 ± 12.99 years, *P* < 0.001), lower proportion of males (60.9%, *P*, 0.001), lower creatinine (1.21 ± 0.81 mg/dL, *P* < 0.001), higher bilirubin (1.21 ± 1.74 mg/dL, *P* < 0.001), less diabetes (5.1%, < 0.001), spent more days in status 2, less pre-transplant support with left and/or right ventricular assist devices. (*P* < 0.001), more likely to receive a concomitant liver transplant (4.9%, *P* < 0.001), lower mean pulmonary artery pressure (25.10 ± 9.74 mmHg, *P* < 0.001), and lower pulmonary capillary wedge pressures (16.91 ± 6.78 mmHg, *P* < 0.001). Compared with other groups, donors for ACHD patients were also younger (28.13 ± 11.23 years, *P* < 0.001), lower proportion of males (64.1%, *P* < 0.001), weighed less (74.99 ± 18.17 kg, *P* < 0.001) and had longer donor heart ischemic times (3.50 ± 1.15, *P* < 0.001).

**Table 1:** Heart failure group characteristics

	Ischemic cardiomyopathy (n = 14,236)	Nonischemic cardiomyopathy (n = 20,676)	Congenital cardiomyopathy (n = 1040)	*P* value
Recipient preoperative features				
Demographics: age	58.37 ± 8.02	50.11 ± 13.08	35.49 ± 12.99	< 0.001
Male	12,408 (87.2%)	14,009 (67.8%)	633 (60.9%)	< 0.001
Weight (kg)	83.89 ± 15.94	81.58 ± 18.61	71.20 ± 18.43	< 0.001
Height (cm)	174.74 ± 8.81	173.46 ± 10.36	169.33 ± 10.92	< 0.001
BMI	27.40 ± 4.44	26.97 ± 5.06	24.67 ± 5.27	< 0.001
Donor/recipient BMI ratio	1.01 ± 0.23	1.02 ± 0.25	1.06 ± 0.26	< 0.001
BSA	2.01 ± 0.22	1.97 ± 0.26	1.82 ± 0.27	< 0.001
Donor/recipient BSA ratio	0.99 ± 0.12	1.01 ± 0.13	1.04 ± 0.14	< 0.001
Comorbidities: creatinine (mg/dL)	1.39 ± 0.83	1.33 ± 0.95	1.21 ± 0.81	< 0.001
Bilirubin (mg/dL)	1.06 ± 1.97	1.14 ± 2.00	1.21 ± 1.74	< 0.001
Diabetes	3888 (27.3%)	3527 (17.1%)	53 (5.1%)	< 0.001
Dialysis	609 (4.3%)	815 (3.9%)	44 (4.2%)	0.288
Cerebrovascular disease	764 (5.4%)	988 (4.8%)	53 (5.1%)	0.047
Malignancy history	813 (5.7%)	1,671 (8.1%)	25 (2.4%)	< 0.001
Presentation acuity: days in status 1A	22.87 ± 46.52	28.09 ± 55.72	28.70 ± 73.33	< 0.001
Days in status 1B	82.93 ± 168.54	87.84 ± 176.33	85.95 ± 176.58	0.034
Days in status 2	96.35 ± 259.41	67.51 ± 209.16	148.75 ± 292.49	< 0.001
Mechanical support: left ventricular assist device	4,146 (29.1%)	6,371 (30.8%)	62 (6.0%)	< 0.001
Right ventricular assist device	23 (0.2%)	33 (0.2%)	7 (0.7%)	0.001
Biventricular support or TAH	328 (2.3%)	759 (3.7%)	17 (1.6%)	< 0.001
Extracorporeal membrane oxygenation	53 (0.4%)	111 (0.5%)	16 (1.5%)	< 0.001
Intra-aortic balloon pump	943 (6.6%)	1,157 (5.6%)	26 (2.5%)	< 0.001
Hemodynamics: cardiac output (L/min)	4.68 ± 1.35	4.45 ± 1.47	4.42 ± 1.30	< 0.001
Systolic pulmonary artery pressure (mmHg)	41.67 ± 14.09	40.87 ± 13.24	37.31 ± 15.00	< 0.001
Diastolic pulmonary artery pressure (mmHg)	19.19 ± 7.99	20.07 ± 8.44	18.21 ± 7.60	< 0.001
Mean pulmonary artery pressure (mmHg)	27.67 ± 9.62	27.99 ± 9.60	25.10 ± 9.74	< 0.001
Pulmonary capillary wedge pressure (mmHg)	18.10 ± 8.22	18.58 ± 8.43	16.91 ± 6.78	< 0.001
Donor: age	32.30 ± 12.00	31.61 ± 11.66	28.13 ± 11.23	< 0.001
Male	10,606 (74.5%)	14,251 (68.9%)	667 (64.1%)	< 0.001
Weight (kg)	82.92 ± 17.87	81.65 ± 19.12	74.99 ± 18.17	< 0.001
Height (cm)	175.10 ± 9.21	173.84 ± 9.78	171.47 ± 10.56	< 0.001
BMI	27.05 ± 5.52	26.99 ± 5.83	25.43 ± 5.41	< 0.001
BSA	2.00 ± 0.24	1.97 ± 0.25	1.88 ± 0.26	< 0.001
Left ventricular ejection fraction (%)	61.58 ± 7.28	61.63 ± 7.08	62.18 ± 7.55	0.034
Heart ischemic time (h)	3.20 ± 1.06	3.12 ± 1.02	3.50 ± 1.15	< 0.001
Coronary artery disease	3,576 (25.1%)	5,213 (25.2%)	193 (18.6%)	< 0.001
Hypertension	1,985 (13.9%)	2,902 (14.0%)	120 (11.5%)	0.076
Diabetes	430 (3.0%)	617 (3.0%)	23 (2.2%)	0.332
Cocaine history	2,242 (15.7%)	3,461 (16.7%)	148 (14.2%)	0.009
Simultaneous transplant: kidney	506 (3.6%)	659 (3.2%)	16 (1.5%)	0.001
Liver	27 (0.2%)	158 (0.8%)	51 (4.9%)	< 0.001

### Survival

**Fig. 1:**
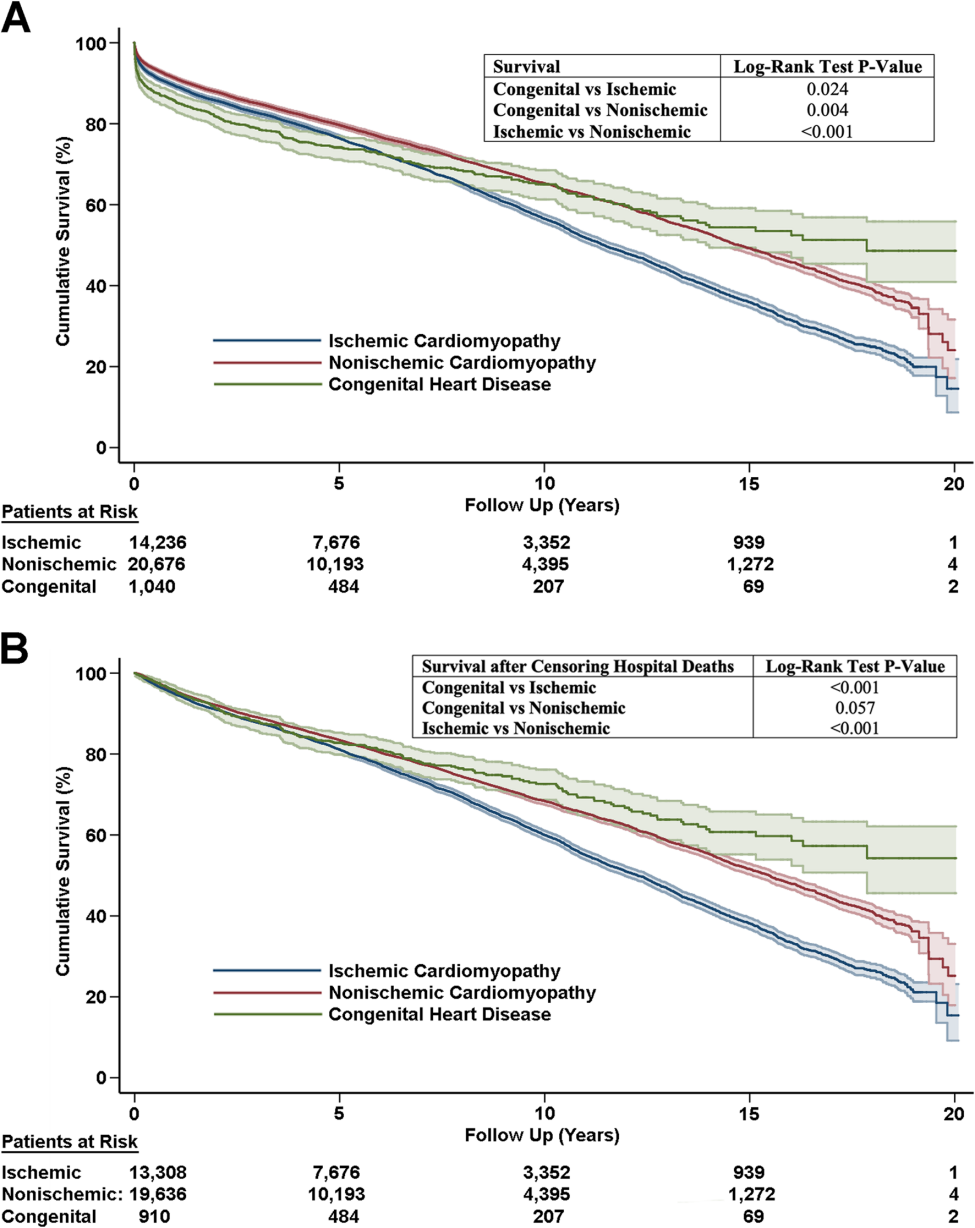
Long term survival of heart failure groups (**A**) and adjusted survival with censoring of in-hospital deaths (**B**)

The mean follow-up for the entire study population (n = 35,952) was 6.20 ± 4.84 years. Univariable analysis for overall survival using Cox Proportional Hazards analysis of recipient, donor and transplant parameters are shown in Additional file 1: Table S1. Subsequent multivariable analysis (Additional file 1: Table S2) showed that ACHD diagnosis for transplantation had survival that was better when compared with ICM (HR = 1.18, *P* = 0.005) and similar when compared to NICM (HR = 0.917, *P* = 0.133). Kaplan–Meier survival analysis also demonstrates early mortality in the ACHD group with its survival curve in an “upward concave” shape (Fig. 1A). Other strong predictors of mortality in the total population included preoperative right ventricular assist device support (HR = 1.50, *P* = 0.041), preoperative biventricular support (HR = 1.20, *P* = 0.001), preoperative extracorporeal membrane oxygenation (HR = 1.60, *P* < 0.001), postoperative stroke (HR = 2.09, *P* < 0.001), postoperative dialysis (HR = 2.95, *P* < 0.001), and prolonged donor heart ischemic time (HR = 1.054, *P* < 0.001). Cumulative survival from 1 to 20 years are shown in Table 2 in each of the three heart failure groups. Impressively, ACHD patients who underwent transplant had a 20 year survival of 47%.

**Table 2:** Survival rates (5,10,15-year) in groups

	ICM (%)	NICM (%)	ACHD (%)
1-year	88.6%	88.6%	83.4%
5-year	75.8%	83.4%	72.3%
10-year	56.1%	68.4%	63.4%
15-year	35.6%	51.6%	53.0%
20-year	13.8%	25.1%	47%
Mean follow-up (years)	6.42 ± 4.89	6.08 ± 4.79	5.67 ± 5.02

*ACHD* adult congenital heart disease, *NICM* non-ischemic cardiomyopathy, *ICM* ischemic cardiomyopathy

Next we censored patients who died in hospital during the initial transplant admission (n = 33,854). Univariable Cox Proportional Hazards analysis was performed in this group (Additional file 1: Table S3) followed by multivariable analysis (Table 3) which confirms that provided the patient survives the transplant admission episode, ACHD had superior survival compared to both ICM (HR = 1.54, *P* < 0.001) and NICM (HR = 1.17, *P* = 0.028). Other notable predictors of adjusted mortality included presence of cerebrovascular disease (HR = 1.11, *P* = 0.026), prior malignancy (HR = 1.12, *P* = 0.006), as well as postoperative stroke (HR = 1.47, *P* < 0.001) and dialysis (HR = 1.71, *P* < 0.001). The divergence in survival is clear from the conditional survival curve (Fig. 1B). ACHD had the best survival followed by NICM. ICM patients had the worse survival (*P* < 0.001, Fig. 1B).

**Table 3:** Multivariable cox proportional hazards analysis (forward and reverse stepwise regression) for long term survival after censoring in-hospital deaths

	B	SE	Wald	*df*	*P* value	HR
Heart failure cause compared with congenital			203.264	2	< 0.001	
Ischemic cardiomyopathy	0.431	0.071	36.567	1	< 0.001	1.539
Nonischemic cardiomyopathy	0.156	0.071	4.805	1	0.028	1.169
Recipient preoperative features						
Age	− 0.002	0.001	4.134	1	0.042	0.998
Body mass index	0.017	0.004	17.948	1	< 0.001	1.017
Cerebrovascular disease	0.103	0.046	4.945	1	0.026	1.109
Malignancy history	0.111	0.040	7.469	1	0.006	1.117
Creatinine (mg/dL)	0.023	0.010	4.864	1	0.027	1.023
Left ventricular assist device	− 0.091	0.026	12.144	1	< 0.001	0.913
Biventricular assist device or total artificial heart	0.110	0.060	3.317	1	0.069	1.116
Systolic pulmonary artery pressure (mmHg)	0.003	0.001	12.804	1	< 0.001	1.003
Donor						
Age	0.009	0.001	133.547	1	< 0.001	1.009
Ischemic time (h)	0.027	0.010	8.059	1	0.005	1.028
Postoperative complications						
Stroke	0.383	0.066	34.013	1	< 0.001	1.467
Dialysis	0.535	0.034	244.521	1	< 0.001	1.708

B = coefficients; SE = asymptotic standard error estimate; Wald = Wald test; df = degrees of freedom; HR = hazard ratio

Given they ACHD patient had excellent survival once out of hospital, we compared the predictors of in-hospital transplant death. Univariable analysis was performed (Additional file 1: Table S4) followed by multivariable evaluation (Table 4) revealed that ACHD was a strong predictor of in-hospital mortality during index transplant admission compared to ICM (HR = 0.54, *P* < 0.001) and NICM (HR = 0.46, *P* < 0.001). Other predictors of mortality included a higher bilirubin (HR = 1.09, *P* < 0.001), preoperative right ventricular assist device (HR = 2.20, *P* = 0.049), preoperative biventricular support (HR = 1.62, *P* < 0.001), preoperative extracorporeal membrane oxygenation (HR = 2.36, *P* < 0.001), postoperative stroke (HR = 4.42, *P* < 0.001), postoperative dialysis (HR = 12.92, *P* < 0.001), and prolonged donor heart ischemic time (*P* = 1.20, *P* < 0.001). Donor O blood type was also associated with higher mortality compared with blood type A (HR = 0.82, *P* = 0.001), B (HR = 0.82, *P* = 0.029), and AB (HR = 0.66, *P* = 0.051). Factors associated with survival included increasing donor/recipient body mass index ratio (HR = 0.51, *P* < 0.001), male donors (HR = 0.85, *P* = 0.011) and donors cocaine use (HR = 0.826, *P* = 0.012).

**Table 4:** Multivariable binary logistic regression for in-hospital death

	B	SE	Wald	*df*	*P* value	HR
Compared with congenital			42.651	2	< 0.001	
Ischemic cardiomyopathy	− 0.619	0.135	21.072	1	< 0.001	0.539
Nonischemic cardiomyopathy	− 0.782	0.128	37.532	1	< 0.001	0.458
Recipient preoperative features						
Age	0.014	0.003	31.887	1	< 0.001	1.015
Height (cm)	− 0.012	0.003	15.946	1	< 0.001	0.988
Body mass index	0.048	0.027	3.079	1	0.079	1.049
Donor/recipient body mass index ratio	− 0.684	0.119	32.795	1	< 0.001	0.505
Creatinine	0.050	0.025	3.989	1	0.046	1.051
Bilirubin	0.088	0.008	132.013	1	< 0.001	1.092
Right ventricular assist device	0.787	0.400	3.867	1	0.049	2.196
Biventricular assist device or total artificial heart	0.484	0.122	15.717	1	< 0.001	1.622
Extracorporeal membrane oxygenation	0.857	0.239	12.844	1	< 0.001	2.356
Systolic pulmonary artery pressure (mmHg)	0.006	0.002	10.165	1	0.001	1.006
Donor						
Age	0.013	0.003	20.835	1	< 0.001	1.013
Male gender	− 0.161	0.064	6.410	1	0.011	0.851
Weight (kg)	0.050	0.019	6.758	1	0.009	1.051
Donor blood type cf. O type			15.598	3	0.001	
A blood type	− 0.194	0.058	11.309	1	0.001	0.824
B Blood Type	− 0.201	0.092	4.768	1	0.029	0.818
AB blood type	− 0.417	0.214	3.801	1	0.051	0.659
Ischemic time (h)	0.181	0.024	57.624	1	< 0.001	1.198
Cocaine use	− 0.191	0.076	6.337	1	0.012	0.826
Postoperative complications						
Stroke	1.485	0.094	249.462	1	< 0.001	4.417
Dialysis	2.559	0.054	2280.401	1	< 0.001	12.922

B = coefficients; SE = asymptotic standard error estimate; Wald = Wald test; df = degrees of freedom; HR = hazard ratio

Cox Proportional hazard analysis also showed recent improvements in survival for ACHD transplantation in 2010–2018 (n = 594) compared to 2000–2009 (n = 446, HR = 0.699, *P* = 0.002). It is important to note that combined heart-liver transplants are performed much more frequently in ACHD compared with Ischemic (5.1% vs. 0.2%, *P* < 0.001) and Nonischemic (5.1% vs. 0.1%, *P* < 0.001) etiologies. However, survival after combined heart-liver transplantation did not differ between ACHD versus ischemic (HR = 1.6, *P* = 0.306) nor nonischemic (HR = 1.3, *P* = 0.461) pathologies.

### Postoperative complications and cardiac retransplantation

**Fig. 2:**
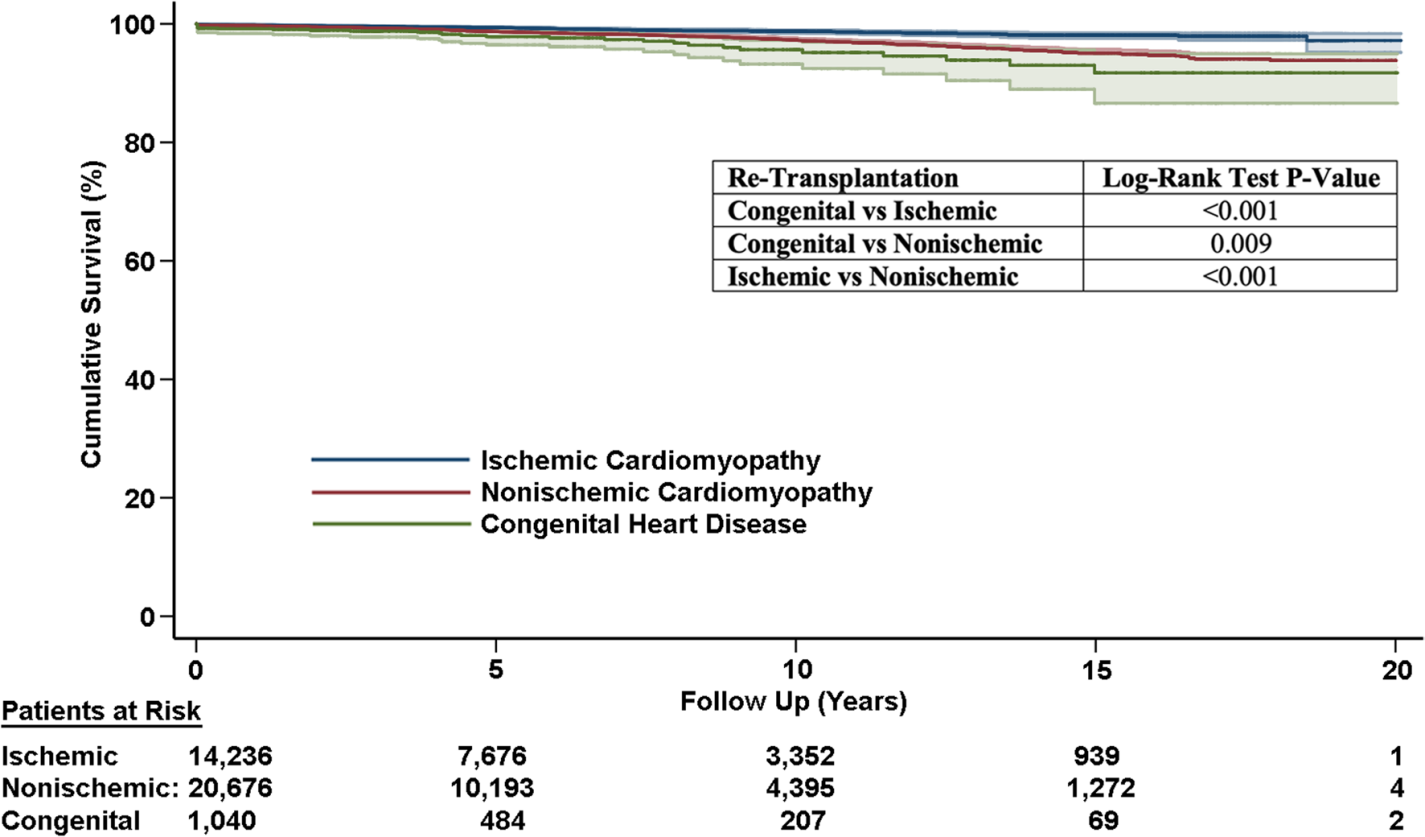
Freedom from retransplantation stratified by underlying diagnosis, either patients with adult congenital heart disease (ACHD), ischemic cardiomyopathy (ICM) or nonischemic cardiomyopathy (NICM)

ACHD had a higher rate of stroke (3.5%, *P* < 0.001) as well as a trend towards higher rates of death from primary graft dysfunction (2.3%, *P* = 0.007, Table 2). ACHD has higher rates of postoperative dialysis compared to NICM (20.6% vs. 10.0%, *P* < 0.001). There was a trend toward higher rates of death from primary graft failure in the ACHD group (2.3%, *P* = 0.071). Interestingly, ICM patients had lower rates of death from acute rejection (1.1%, *P* < 0.001). ACHD patients had the highest rate of retransplantation (2.8%, *P* < 0.001) followed by NICM (1.8%) and then finally ICM (0.9%). Kaplan-Meieer curve for retranplantation is shown in Fig. 2.

## Discussion

Heart transplantation in ACHD patients presents unique challenges. In the 1950s, only 30% of births with congenital heart disease survived beyond infancy [9]. Currently, about 85–90% survive into adolescence and adulthood [4, 10]. It is estimated that about 10–20% of patients with congenital heart disease will need heart tranaplantation at some point although it is likely high in the contemporary setting [11, 12]. Most recipients with ACHD also have prior cardiac surgery and complex anatomy requiring obligate reconstructive surgery in more than 75% of these individuals operation. These may include extensive reconstructions of the aortic arch, vena cava, and/or pulmonary arteries [13]. Redo-sternotomies and additional procedural complexity contributes to longer cardiopulmonary bypass times with associated operative coagulopathy [8]. Additional factors inpacting early outcomes in ACHD patients include single ventricle physiology, more previous cardiac surgeries, presence of protein-losing enteropathy, and sensitization to alloantigens [14].

Not surprisingly, ACHD patients have a distinct profile consisting of different baseline characteristics than ICM and NICM groups. ACHD patients have favorable features such as tendency to be younger, with better renal function, lower pulmonary artery pressure and less ventricular assist device use pre-transplant. However, this is negatively counteracted by the greater operative complexity and more liver dysfunction as indicated by higher bilirubin. Although younger donor hearts were used in the ACHD population, these were subjected to a longer ischemic time. This risk profile with greater anatomical complexity is consistent with our finding that early mortality is higher in the ACHD group. First year survival was worse for ACHD (83.4%) compared with ICM (88.6%) and NICM (88.6%). Based on our conditional survival with censoring of deaths during the index transplant admission (Fig. 1B), ACHD patients benefit from much better long term survival provided they survive the initial transplant surgery and admission. Compared to ACHD the relative risk of mortality was 1.5 times for ICM and 1.2 times for NICM. Not surprisingly, long term mortality was highly associated with the occurrence of postoperative stroke (HR = 1.47) and postoperative dialysis (HR = 1.71). Given the younger age of ACHD patients, this group is much more likely to undergo retransplantation (Fig. 2). Other groups have also noted the initial surgical risks but increased long term transplant survival of ACHD patients given their young age [15–18]. Our study further defines the need to anticipate future cardiac retransplantation and the need for close management to optimize the graft survival duration in this population (Table 5).

**Table 5:** Post-transplant outcomes of heart failure groups

	Ischemic cardiomyopathy (n = 14,236)	Nonischemic cardiomyopathy (n = 20,676)	Congenital cardiomyopathy (n = 1040)	*P* value
Postop stroke	419 (2.9%)	459 (2.2%)	36 (3.5%)	< 0.001
Postop dialysis	1513 (19.6%)	2059 (10.0%)	214 (20.6%)	< 0.001
Postop pacemaker	458 (3.2%)	673 (3.3%)	28 (2.7%)	0.604
Death from primary graft failure	225 (1.6%)	300 (1.5%)	24 (2.3%)	0.071
Death from hyperacute rejection	19 (0.1%)	17 (0.1%)	2 (0.2%)	0.240
Death from acute rejection	157 (1.1%)	447 (2.2%)	24 (2.3%)	< 0.001
Death from chronic rejection with graft vasculopathy	244 (1.7%)	389 (1.9%)	18 (1.7%)	0.505

Given these findings, we determined the the factors influencing operative or in-hospital survival. ACHD was highly associated with index transplant in-hospital mortality compared with ICM (HR = 0.54) and NICM (HR = 0.46). Other prominent factors associated with mortality included mechanical circulatory support such as right ventricular assist device use (HR = 2.20), biventricular support (HR = 1.62) and extracorporeal membrane oxygenation (HR = 2.36). Again the postoperative stroke (HR = 4.42) and dialysis (HR = 12.92) were important drivers of mortality. The higher incidence of stroke in ACHD may reflect the presence of residual shunts and thromboembolic substrates such as venous collaterals. Preemptive closure of shunts via transcatheter approaches and early cross clamp of the aorta during the transplant may help minimize stroke risk.

The longer donor heart ischemic time in the ACHD also translated into more deaths from primary graft dysfunction in this group. This suggests that the recent availability of commercial normothermic or hypothermic machine perfusion used for donor organ transport may mitigate against the effects of prolonged ischemic times in this ACHD group of patients requiring complex operative intervention and incurring longer operative times [19,20]. The higher incidence of death from acute rejection in the ACHD versus ischemic heart disease group as well as more combined heart-liver transplantation in the ACHD population may also have contributed to reduced short term survival. However, our demonstrated improvements in ACHD survival over time era likely reflects advancements in surgical expertise and medical management (Additional file 1).

Study limitations are that the UNOS database does not provide sufficient granularity to identify ACHD subtypes such as single ventricle physiology, Epstein’s Anomaly, or transposition of great arteries. Furthermore, the population of ACHD undergoing heart transplantation is relatively small and limits the power of the study to perform more detailed analysis.

In conclusion, we found that although adult ACHD patients had a higher early mortality rate after heart transplantation, and they had better long-term survival compared to non-ACHD recipients. This can be explained by the higher operative mortality given physiologcal and anatomical complexity of ACHD patients but a favorable longer term survival given their younger age provided operative survival was achieved. The longer donor heart ischemic times in ACHD resulting primary graft dysfunction was a major driver of the operative risk. More liberal use of modern normothermic perfusion transport techniques may mitigate the risk of primary graft dysfunction in this population.

### Supplementary Information

**Additional file 1:** Multivariable and univariate cox proportional hazards analysis for long term survival and univariate binary logistic regression for in-hospital death.

## Data Availability

The data underlying this article will be shared on reasonable request to the corresponding author.
