# Supplementary material for: Determinants of survival following heart transplantation in adults with congenital heart disease
Source: J Cardiothorac Surg. 2024 Feb 10;19:83. doi: 10.1186/s13019-024-02509-0 (PMC10858543; doi:10.1186/s13019-024-02509-0)
Supplement: Supplementary file 1 — Additional file 1: Multivariable and univariate cox proportional hazards analysis for long term survival and univariate binary logistic regression for in-hospital death. [file 13019_2024_2509_MOESM1_ESM.docx]

**Supplementary Table 1. Univariate Cox Proportional Hazards Analysis for Long Term Survival. B = Coefficients; SE = Asymptotic Standard Error Estimate; Wald = Wald test; df = Degrees of Freedom; HR = Hazard Ratio.**

|  | **B** | **SE** | **Wald** | **df** | **P Value** | **HR** |
| --- | --- | --- | --- | --- | --- | --- |
| Heart Failure Cause Compared with Congenital |  |  | 242.339 | 2 | <0.001 |  |
| Ischemic Cardiomyopathy | 0.127 | 0.056 | 5.154 | 1 | 0.023 | 1.135 |
| Nonischemic Cardiomyopathy | -0.158 | 0.056 | 8.009 | 1 | 0.005 | 0.854 |
| Recipient |  |  |  |  |  |  |
| Age | 0.008 | 0.001 | 115.534 | 1 | <0.001 | 1.008 |
| Male gender | 0.039 | 0.021 | 3.455 | 1 | 0.063 | 1.040 |
| Height (cm) | -0.003 | 0.001 | 9.419 | 1 | 0.002 | 0.997 |
| Weight (kg) | 0.003 | 0.001 | 25.635 | 1 | <0.001 | 1.003 |
| BMI | 0.016 | 0.002 | 68.625 | 1 | <0.001 | 1.016 |
| BMI Donor/Recipient | -0.141 | 0.038 | 13.793 | 1 | <0.001 | 0.868 |
| BSA | 0.129 | 0.037 | 12.385 | 1 | <0.001 | 1.137 |
| BSA Donor/Recipient | -0.131 | 0.074 | 3.147 | 1 | 0.076 | 0.877 |
| Diabetes | 0.188 | 0.023 | 65.378 | 1 | <0.001 | 1.207 |
| Dialysis pretxp | 0.407 | 0.041 | 97.598 | 1 | <0.001 | 1.502 |
| Cerebrovascular disease | 0.140 | 0.041 | 11.393 | 1 | 0.001 | 1.150 |
| Prior malignancy | 0.106 | 0.036 | 8.655 | 1 | 0.003 | 1.112 |
| Cr | 0.079 | 0.007 | 134.101 | 1 | <0.001 | 1.082 |
| Bilirubin | 0.028 | 0.003 | 100.107 | 1 | <0.001 | 1.028 |
| PreTxp LVAD | -0.049 | 0.023 | 4.623 | 1 | 0.032 | 0.953 |
| PreTxp RVAD | 0.535 | 0.196 | 7.414 | 1 | 0.006 | 1.707 |
| PreTxp BIVAD or TAH | 0.259 | 0.050 | 26.604 | 1 | <0.001 | 1.296 |
| Status 1A time | 0.001 | <0.001 | 8.243 | 1 | 0.004 | 1.001 |
| Status 1B time | <0.001 | <0.001 | 0.014 | 1 | 0.905 | 1.000 |
| Status 2 time | <0.001 | <0.001 | 2.428 | 1 | 0.119 | 1.000 |
| ECMO PreTxp | 0.639 | 0.117 | 30.020 | 1 | <0.001 | 1.895 |
| IABP PreTxp | 0.104 | 0.039 | 7.161 | 1 | 0.007 | 1.109 |
| Cardiac Output (L/min) | 0.006 | 0.006 | 1.035 | 1 | 0.309 | 1.006 |
| PA systolic (mmHg) | 0.005 | 0.001 | 51.686 | 1 | <0.001 | 1.005 |
| PA Diastolic (mmHg) | 0.006 | 0.001 | 28.132 | 1 | <0.001 | 1.006 |
| PA mean (mmHg) | 0.006 | 0.001 | 36.071 | 1 | <0.001 | 1.006 |
| PCWP (mmHg) | 0.003 | 0.001 | 7.656 | 1 | 0.006 | 1.003 |
| Postop Stroke | 0.990 | 0.045 | 486.382 | 1 | <0.001 | 2.692 |
| Postop Dialysis | 1.160 | 0.024 | 2398.550 | 1 | <0.001 | 3.190 |
| Postop Pacemaker | 0.056 | 0.049 | 1.294 | 1 | 0.255 | 1.058 |
| Simultaneous Kidney Txp | 0.026 | 0.056 | 0.212 | 1 | 0.645 | 1.026 |
| Simultaneous Liver Txp | -0.086 | 0.133 | 0.422 | 1 | 0.516 | 0.917 |
| Donor |  |  |  |  |  |  |
| Age | 0.011 | 0.001 | 224.639 | 1 | <0.001 | 1.011 |
| Male Gender | -0.042 | 0.020 | 4.568 | 1 | 0.033 | 0.958 |
| Height (cm) | -0.001 | 0.001 | 0.340 | 1 | 0.560 | 0.999 |
| Weight (kg) | 0.001 | <0.001 | 3.878 | 1 | 0.049 | 1.001 |
| BMI | 0.004 | 0.002 | 5.177 | 1 | 0.023 | 1.004 |
| BSA | 0.062 | 0.037 | 2.885 | 1 | 0.089 | 1.064 |
| Donor Blood Type cf. O Type |  |  | 6.544 | 3 | 0.088 |  |
| A Blood Type | -0.046 | 0.020 | 5.535 | 1 | 0.019 | 0.955 |
| B Blood Type | -0.022 | 0.030 | 0.528 | 1 | 0.468 | 0.978 |
| AB Blood Type | -0.083 | 0.065 | 1.664 | 1 | 0.197 | 0.920 |
| Ischemic Time (hours) | 0.073 | 0.009 | 71.396 | 1 | <0.001 | 1.075 |
| LVEF | -0.001 | 0.001 | 0.989 | 1 | 0.320 | 0.999 |
| Coronary Artery Disease on Angio | 0.156 | 0.021 | 54.629 | 1 | <0.001 | 1.169 |
| Hypertension | 0.138 | 0.026 | 28.748 | 1 | <0.001 | 1.148 |
| Diabetes | 0.127 | 0.054 | 5.567 | 1 | 0.018 | 1.135 |
| Cocaine Use | 0.028 | 0.026 | 1.163 | 1 | 0.281 | 0.973 |

**Supplementary Table 2. Multivariable Cox Proportional Hazards Analysis for Long Term Survival. B = Coefficients; SE = Asymptotic Standard Error Estimate; Wald = Wald test; df = Degrees of Freedom; HR = Hazard Ratio.**

|  | **B** | **SE** | **Wald** | **df** | **P Value** | **HR** |
| --- | --- | --- | --- | --- | --- | --- |
| Heart Failure Cause Compared with Congenital |  |  | 166.632 | 2 | 0.000 |  |
| Ischemic Cardiomyopathy | 0.169 | 0.060 | 7.961 | 1 | 0.005 | 1.184 |
| Nonischemic Cardiomyopathy | -0.087 | 0.058 | 2.253 | 1 | 0.133 | 0.917 |
| Recipient Preoperative Features |  |  |  |  |  |  |
| Age | 0.004 | 0.001 | 17.251 | 1 | 0.000 | 1.004 |
| Height (cm) | 0.036 | 0.011 | 10.356 | 1 | 0.001 | 1.037 |
| Weight (kg) | 0.034 | 0.011 | 9.264 | 1 | 0.002 | 1.034 |
| Body mass index | 0.053 | 0.017 | 9.732 | 1 | 0.002 | 1.055 |
| Diabetes | 0.071 | 0.024 | 8.411 | 1 | 0.004 | 1.073 |
| Dialysis | -0.097 | 0.047 | 4.290 | 1 | 0.038 | 0.908 |
| Cerebrovascular disease | 0.086 | 0.042 | 4.272 | 1 | 0.039 | 1.090 |
| Prior malignancy | 0.089 | 0.036 | 5.914 | 1 | 0.015 | 1.093 |
| Creatinine | 0.020 | 0.010 | 4.044 | 1 | 0.044 | 1.020 |
| Bilirubin | 0.027 | 0.003 | 83.653 | 1 | <0.001 | 1.027 |
| Left ventricular assist device | -0.093 | 0.025 | 14.117 | 1 | <0.001 | 0.911 |
| Right ventricular assist device | 0.403 | 0.197 | 4.172 | 1 | 0.041 | 1.496 |
| Biventricular assist device or Total artificial heart | 0.180 | 0.052 | 11.915 | 1 | 0.001 | 1.197 |
| Extracorporeal membrane oxygenation | 0.472 | 0.118 | 16.102 | 1 | <0.001 | 1.603 |
| Systolic pulmonary artery pressure (mmHg) | 0.003 | 0.001 | 8.347 | 1 | 0.004 | 1.003 |
| Diastolic pulmonary artery pressure (mmHg) | 0.005 | 0.002 | 4.045 | 1 | 0.044 | 1.005 |
| Pulmonary capillary wedge pressure (mmHg) | -0.008 | 0.002 | 17.002 | 1 | 0.000 | 0.992 |
| Donor |  |  |  |  |  |  |
| Age | 0.010 | 0.001 | 113.479 | 1 | <0.001 | 1.010 |
| BMI | -0.005 | 0.002 | 7.503 | 1 | 0.006 | 0.995 |
| Donor Blood Type c.f. O Type |  |  | 14.023 | 3 | 0.003 |  |
| A Blood Type | -0.069 | 0.020 | 11.968 | 1 | 0.001 | 0.933 |
| B Blood Type | -0.001 | 0.031 | 0.001 | 1 | 0.982 | 0.999 |
| AB Blood Type | -0.094 | 0.065 | 2.094 | 1 | 0.148 | 0.910 |
| Ischemic Time (hours) | 0.053 | 0.009 | 36.406 | 1 | <0.001 | 1.054 |
| Coronary Artery Disease on Angio | -0.058 | 0.026 | 4.762 | 1 | 0.029 | 0.944 |
| Postoperative Complications |  |  |  |  |  |  |
| Stroke | 0.739 | 0.046 | 262.405 | 1 | <0.001 | 2.094 |
| Dialysis | 1.083 | 0.025 | 1830.593 | 1 | <0.001 | 2.953 |

**Supplementary Table 3. Univariate Cox Proportional Hazards Analysis for Long Term Survival after Censoring In-Hospital Deaths. B = Coefficients; SE = Asymptotic Standard Error Estimate; Wald = Wald test; df = Degrees of Freedom; HR = Hazard Ratio.**

|  | **B** | **SE** | **Wald** | **df** | **P Value** | **HR** |
| --- | --- | --- | --- | --- | --- | --- |
| Heart Failure Cause Compared with Congenital |  |  | 222.674 | 2 | .000 |  |
| Ischemic Cardiomyopathy | 0.425 | 0.071 | 36.161 | 1 | .000 | 1.529 |
| Nonischemic Cardiomyopathy | 0.137 | 0.071 | 3.763 | 1 | .052 | 1.147 |
| Recipient |  |  |  |  |  |  |
| Age | 0.008 | 0.001 | 79.518 | 1 | <0.001 | 1.008 |
| Male gender | 0.059 | 0.023 | 6.395 | 1 | 0.011 | 1.061 |
| Height (cm) | -0.001 | 0.001 | 0.292 | 1 | 0.589 | 0.999 |
| Weight (kg) | 0.003 | 0.001 | 23.044 | 1 | <0.001 | 1.003 |
| BMI | 0.013 | 0.002 | 41.166 | 1 | <0.001 | 1.013 |
| BMI Donor/Recipient | -0.067 | 0.041 | 2.660 | 1 | 0.103 | 0.935 |
| BSA | 0.157 | 0.040 | 15.280 | 1 | <0.001 | 1.170 |
| BSA Donor/Recipient | -0.029 | 0.081 | 0.126 | 1 | 0.723 | 0.972 |
| Diabetes | 0.235 | 0.026 | 82.429 | 1 | <0.001 | 1.265 |
| Dialysis pretxp | 0.221 | 0.050 | 19.535 | 1 | <0.001 | 1.247 |
| Cerebrovascular disease | 0.130 | 0.046 | 7.827 | 1 | 0.005 | 1.138 |
| Prior malignancy | 0.102 | 0.040 | 6.392 | 1 | 0.011 | 1.107 |
| Creatinine | 0.057 | 0.009 | 41.027 | 1 | <0.001 | 1.058 |
| Bilirubin | -0.002 | 0.006 | 0.176 | 1 | 0.675 | 0.998 |
| PreTxp LVAD | -0.075 | 0.026 | 8.523 | 1 | 0.004 | 0.928 |
| PreTxp RVAD | -0.172 | 0.316 | 0.297 | 1 | 0.586 | 0.842 |
| PreTxp BIVAD or TAH | 0.120 | 0.060 | 4.023 | 1 | 0.045 | 1.127 |
| Status 1A time | <0.001 | <0.001 | 0.068 | 1 | 0.794 | 1.000 |
| Status 1B time | <0.001 | <0.001 | 0.409 | 1 | 0.522 | 1.000 |
| Status 2 time | <0.001 | <0.001 | 0.745 | 1 | 0.388 | 1.000 |
| ECMO PreTxp | -0.033 | 0.186 | 0.031 | 1 | 0.860 | 0.968 |
| IABP PreTxp | 0.075 | 0.044 | 2.925 | 1 | 0.087 | 1.078 |
| Cardiac Output (L/min) | 0.011 | 0.007 | 2.757 | 1 | 0.097 | 1.011 |
| PA systolic (mmHg) | 0.004 | 0.001 | 26.643 | 1 | <0.001 | 1.004 |
| PA Diastolic (mmHg) | 0.004 | 0.001 | 12.657 | 1 | <0.001 | 1.004 |
| PA mean (mmHg) | 0.004 | 0.001 | 18.251 | 1 | <0.001 | 1.004 |
| PCWP (mmHg) | 0.002 | 0.001 | 2.517 | 1 | 0.113 | 1.002 |
| Postop Stroke | 0.475 | 0.065 | 52.746 | 1 | <0.001 | 1.607 |
| Postop Dialysis | 0.577 | 0.033 | 300.182 | 1 | <0.001 | 1.781 |
| Postop Pacemaker | 0.085 | 0.053 | 2.526 | 1 | 0.112 | 1.089 |
| Simultaneous Kidney Txp | -0.065 | 0.065 | 0.982 | 1 | 0.322 | 0.937 |
| Simultaneous Liver Txp | -0.114 | 0.153 | 0.560 | 1 | 0.454 | 0.892 |
| Donor |  |  |  |  |  |  |
| Age | 0.010 | 0.001 | 169.161 | 1 | <0.001 | 1.011 |
| Male Gender | 0.003 | 0.022 | 0.014 | 1 | 0.907 | 1.003 |
| Height (cm) | 0.001 | 0.001 | 1.835 | 1 | 0.176 | 1.001 |
| Weight (kg) | 0.002 | 0.001 | 12.365 | 1 | <0.001 | 1.002 |
| BMI | 0.005 | 0.002 | 9.502 | 1 | 0.002 | 1.005 |
| BSA | 0.141 | 0.040 | 12.337 | 1 | <0.001 | 1.152 |
| Donor Blood Type cf. O Type |  |  | 3.479 | 3 | 0.324 |  |
| A Blood Type | -0.031 | 0.022 | 2.039 | 1 | 0.153 | 0.970 |
| B Blood Type | 0.025 | 0.033 | 0.563 | 1 | 0.453 | 1.025 |
| AB Blood Type | -0.020 | 0.069 | 0.081 | 1 | 0.776 | 0.981 |
| Ischemic Time (hours) | 0.035 | 0.010 | 13.132 | 1 | <0.001 | 1.035 |
| LVEF | -0.001 | 0.001 | 0.565 | 1 | 0.452 | 0.999 |
| Coronary Artery Disease on Angio | 0.148 | 0.024 | 39.825 | 1 | <0.001 | 1.160 |
| Hypertension | 0.148 | 0.028 | 27.372 | 1 | <0.001 | 1.160 |
| Diabetes | 0.137 | 0.060 | 5.261 | 1 | 0.022 | 1.147 |
| Cocaine Use | 0.002 | 0.028 | 0.006 | 1 | 0.940 | 1.002 |

**Supplementary Table 4. Univariate Binary Logistic Regression for In-Hospital Death. B = Coefficients; SE = Asymptotic Standard Error Estimate; Wald = Wald test; df = Degrees of Freedom; HR = Hazard Ratio.**

|  | **B** | **SE** | **Wald** | **df** | **P Value** | **HR** |
| --- | --- | --- | --- | --- | --- | --- |
| Heart Failure Cause Compared with Congenital |  |  | 97.212 | 2 | <0.001 |  |
| Ischemic Cardiomyopathy | -0.713 | 0.105 | 45.860 | 1 | <0.001 | 0.490 |
| Nonischemic Cardiomyopathy | -0.971 | 0.104 | 86.418 | 1 | <0.001 | 0.379 |
| Recipient |  |  |  |  |  |  |
| Age | 0.012 | 0.002 | 35.807 | 1 | <0.001 | 1.012 |
| Male gender | -0.080 | 0.054 | 2.166 | 1 | 0.141 | 0.923 |
| Height (cm) | -0.014 | 0.002 | 34.444 | 1 | <0.001 | 0.986 |
| Weight (kg) | 0.001 | 0.001 | 1.249 | 1 | 0.264 | 1.001 |
| BMI | 0.024 | 0.005 | 24.712 | 1 | <0.001 | 1.024 |
| BMI Donor/Recipient | -0.537 | 0.104 | 26.391 | 1 | <0.001 | 0.585 |
| BSA | -0.045 | 0.095 | 0.223 | 1 | 0.637 | 0.956 |
| BSA Donor/Recipient | -0.684 | 0.199 | 11.758 | 1 | 0.001 | 0.505 |
| Diabetes | -0.045 | 0.059 | 0.580 | 1 | 0.446 | 0.956 |
| Dialysis pretxp | 1.023 | 0.083 | 152.288 | 1 | <0.001 | 2.781 |
| Cerebrovascular disease | 0.150 | 0.103 | 2.149 | 1 | 0.143 | 1.162 |
| Prior malignancy | 0.125 | 0.089 | 1.956 | 1 | 0.162 | 1.133 |
| Cr | 0.169 | 0.017 | 100.959 | 1 | <0.001 | 1.184 |
| Bilirubin | 0.109 | 0.008 | 202.849 | 1 | <0.001 | 1.115 |
| PreTxp LVAD | 0.035 | 0.052 | 0.447 | 1 | 0.504 | 1.035 |
| PreTxp RVAD | 1.465 | 0.322 | 20.730 | 1 | <0.001 | 4.327 |
| PreTxp BIVAD or TAH | 0.715 | 0.104 | 47.091 | 1 | <0.001 | 2.045 |
| Status 1A time | 0.002 | <0.001 | 24.439 | 1 | <0.001 | 1.002 |
| Status 1B time | 0.000 | <0.001 | 1.535 | 1 | 0.215 | 1.000 |
| Status 2 time | 0.000 | <0.001 | 1.533 | 1 | 0.216 | 1.000 |
| ECMO PreTxp | 1.464 | 0.192 | 58.156 | 1 | <0.001 | 4.322 |
| IABP PreTxp | 0.239 | 0.092 | 6.759 | 1 | 0.009 | 1.270 |
| Cardiac Output (L/min) | -0.017 | 0.017 | 0.986 | 1 | 0.321 | .983 |
| PA systolic (mmHg) | 0.009 | 0.002 | 31.923 | 1 | <0.001 | 1.010 |
| PA Diastolic (mmHg) | 0.013 | 0.003 | 22.799 | 1 | <0.001 | 1.013 |
| PA mean (mmHg) | 0.011 | 0.002 | 21.196 | 1 | <0.001 | 1.011 |
| PCWP (mmHg) | 0.009 | 0.003 | 10.679 | 1 | 0.001 | 1.009 |
| Postop Stroke | 2.073 | 0.078 | 702.445 | 1 | <0.001 | 7.947 |
| Postop Dialysis | 2.752 | 0.051 | 2914.904 | 1 | <0.001 | 15.667 |
| Postop Pacemaker | -0.136 | 0.143 | 0.903 | 1 | 0.342 | 0.873 |
| Simultaneous Kidney Txp | 0.324 | 0.117 | 7.645 | 1 | 0.006 | 1.382 |
| Simultaneous Liver Txp | -0.212 | 0.324 | 0.430 | 1 | 0.512 | 0.809 |
| Donor |  |  |  |  |  |  |
| Age | 0.015 | 0.002 | 59.664 | 1 | <0.001 | 1.015 |
| Male Gender | -0.257 | 0.050 | 26.079 | 1 | <0.001 | 0.774 |
| Height (cm) | -0.011 | 0.002 | 18.620 | 1 | <0.001 | 0.990 |
| Weight (kg) | -0.004 | 0.001 | 10.989 | 1 | 0.001 | 0.996 |
| BMI | -0.007 | 0.004 | 3.078 | 1 | 0.079 | 0.993 |
| BSA | -0.383 | 0.097 | 15.413 | 1 | <0.001 | 0.682 |
| Donor Blood Type cf. O Type |  |  | 18.555 | 3 | <0.001 |  |
| A Blood Type | -0.147 | 0.052 | 8.104 | 1 | 0.004 | 0.863 |
| B Blood Type | -0.239 | 0.084 | 8.120 | 1 | 0.004 | 0.788 |
| AB Blood Type | -0.544 | 0.202 | 7.248 | 1 | 0.007 | 0.580 |
| Ischemic Time (hours) | 0.255 | 0.021 | 147.140 | 1 | <0.001 | 1.290 |
| LVEF | -0.004 | 0.003 | 1.426 | 1 | 0.232 | 0.996 |
| Coronary Artery Disease on Angio | 0.182 | 0.053 | 11.793 | 1 | 0.001 | 1.199 |
| Hypertension | 0.116 | 0.066 | 3.078 | 1 | 0.079 | 1.123 |
| Diabetes | 0.068 | 0.136 | 0.247 | 1 | 0.619 | 1.070 |
| Cocaine Use | -0.204 | 0.069 | 8.837 | 1 | 0.003 | .815 |
